# Rhabdomyosarcoma of the uterus in an adult patient with osteopetrosis: a case report

**DOI:** 10.1186/s13256-021-03172-y

**Published:** 2021-11-28

**Authors:** Soheila Aminimoghaddam, Ali Rahbari, Roghayeh Pourali

**Affiliations:** 1 Department of Obstetrics and Gynecology, Firoozgar Hospital, Iran University of Medical Sciences, Tehran, Iran; 2 Department of Pathology, Jam Hospital, Tehran, Iran; 3 Department of Obstetrics and Gynecology, Jam Hospital, Fajr Street, Motahari Street, 1588657915 Tehran, Iran

**Keywords:** Rhabdomyosarcoma, Uterus, Sarcoma

## Abstract

**Background:**

Uterine sarcoma accounts for 3–7% of uterine malignant neoplasms. It is more aggressive than epithelial neoplasms, and patients have a poor prognosis. Rhabdomyosarcoma is classified as a heterologous uterine sarcoma. It is the most common soft tissue malignancy in children while rare in adults. In young patients, the majority of genital tract rhabdomyosarcomas occur in vagina; however, the most common site of gynecologic rhabdomyosarcoma is cervix followed by uterine corpus, in adults. Uterine corpus rhabdomyosarcoma is rare in adults. Diagnosis of pure rhabdomyosarcoma in uterus involves widespread and perfect sampling as well as precise histopathological evaluation to uncover any epithelial component.

**Case presentation:**

Here we report a case of pure rhabdomyosarcoma of uterine corpus in a 60-year-old Iranian postmenopausal female who had osteopetrosis, presenting with 8-month heavy vaginal bleeding and a protruding cervical mass. She is alive on 18-month follow-up after treatment.

**Conclusions:**

Rhabdomyosarcoma of uterine corpus is rare in adults. Diagnosis of pure rhabdomyosarcoma in uterus involves widespread and perfect sampling as well as precise histopathological evaluation to uncover any epithelial component. Treatment options in adult gynecological rhabdomyosarcoma are based on studies in younger patients, and more studies may help us choose the best approach for improving outcome.

## Introduction

Rhabdomyosarcoma (RMS) is an aggressive tumor that tends to develop in children and younger patients [1]. A large majority of genital tract RMSs occurs in infants’ and adolescents’ vagina [2]. RMS of uterine corpus either occurs as a component of biphasic uterine tumor (adenosarcoma or carcinosarcoma) or can be a pure heterologous tumor [3, 4]. Pure uterine RMS is rare in adult patients and difficult to diagnose. Accurate diagnosis of these tumors depends on precise histopathological evaluation [5]. We hereby report a case of pure RMS of uterine corpus in a 60-year-old postmenopausal female who had osteopetrosis. There is limited experience with RMS management in adults. Older age, widespread disease at the time of diagnosis, and unfavorable histologic variants such as pleomorphic and alveolar led to poor outcomes in adult patients despite multidisciplinary approach. Reviewing rare cases can result in more rapid diagnosis and improved treatment and, consequently, best possible survival rate.

## Case report

A 60-year-old Iranian female patient attended our emergency department with complaints of heavy vaginal bleeding during last 8 months. In her medical history, she had three vaginal deliveries, diabetes mellitus, depression, and osteopetrosis. Her menopause started at 50 years of age. She did not report any history of sexually transmitted infection or receiving hormonal medications. Her medications included metformin (500 mg twice a day), sertraline (50 mg daily), vitamin D (1000 mg daily), and calcium (500 mg daily). She had undergone surgery twice over the last 10 years, due to fracture of lower extremities. Her family history was unremarkable.

**Fig. 1:**
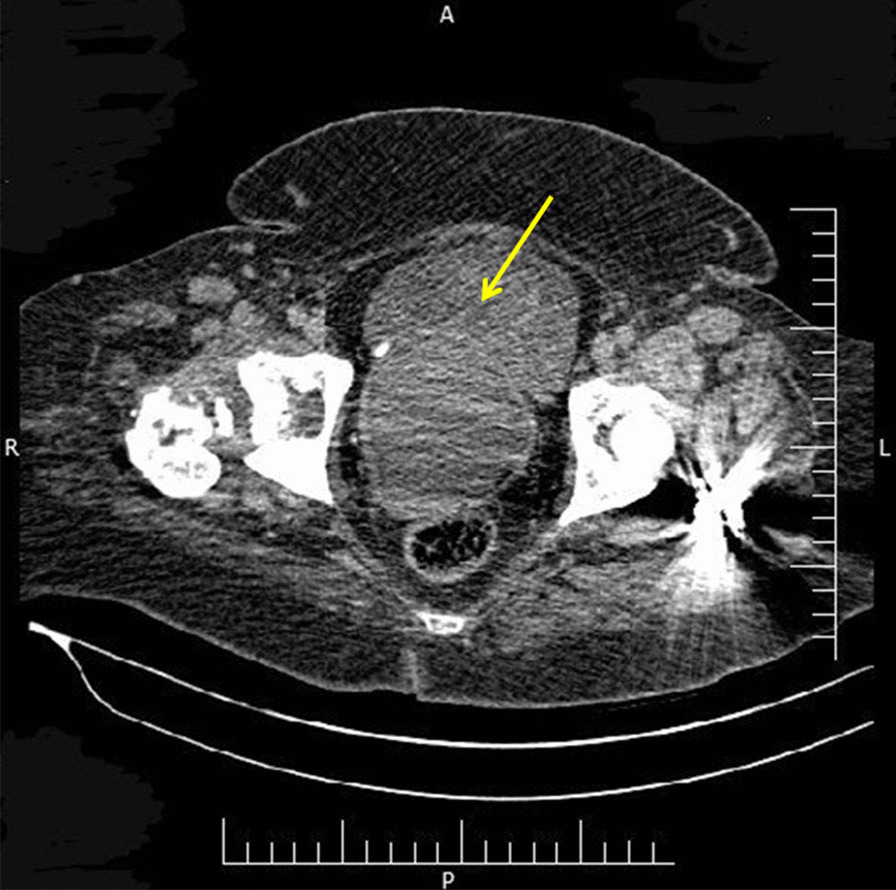
A large mass (yellow arrow) in posterior wall of uterus in axial-view CT scan

She was admitted to our hospital. Hemoglobin level was 6 g/dl at the time of admission. The other hematologic and biochemistry tests revealed no significant problems. On pelvic examination, which was performed under anesthesia, we found a remarkably enlarged uterus and a 10-cm soft hemorrhagic mass originating from posterior part of cervix and protruded into vagina. Excisional biopsy was performed. Abdominopelvic computerized tomography (CT) scans revealed a large uterus, a 15-cm pelvic mass in posterior wall of uterus (Fig. 1), and enlarged pelvic lymph nodes. Chest CT scan and whole-body bone scan were normal. Pathology reported the cervical mass as a high-grade sarcoma in favor of pleomorphic rhabdomyosarcoma.

**Fig. 2:**
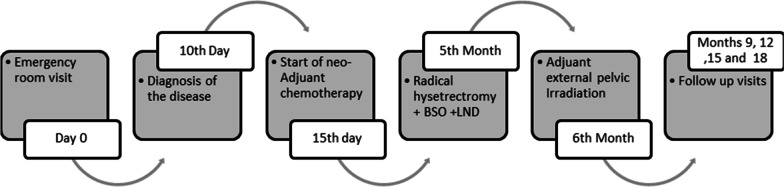
Patient medical history timeline and clinical case presentation

After a multidisciplinary meeting, the patient was treated with multiagent neoadjuvant chemotherapy. We prescribed intravenous vincristine (2 mg), actinomycin-D (1 g), and cyclophosphamide (1200 mg) (VAC) every 3 weeks for six cycles. Delayed radical hysterectomy, bilateral salpingoophorectomy (BSO), and pelvic lymph node dissection (PLND) were performed due to large bulky tumor and partial response to initial chemotherapy. Her postoperative condition was appropriate, and she was discharged 3 days after surgery. Following discharge, external pelvic irradiation (total dose of 5040 cGy in 28 fractions) was performed in 6 weeks. No complications, such as radiation cystitis or proctitis, occurred. She signed an informed consent form and gave us permission to report her disease. She was followed up every 3 months and was free of disease 18 months after treatment (Figure 2).

## Pathology report

**Fig. 3:**
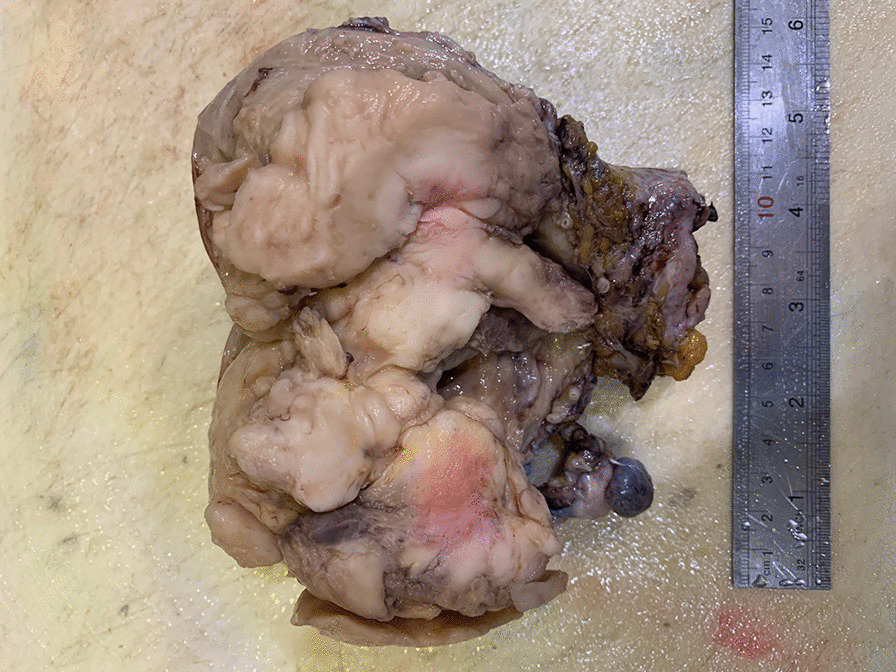
Gross tumor showing cream-colored fleshy mass filling entire endometrial cavity

**Fig. 4:**
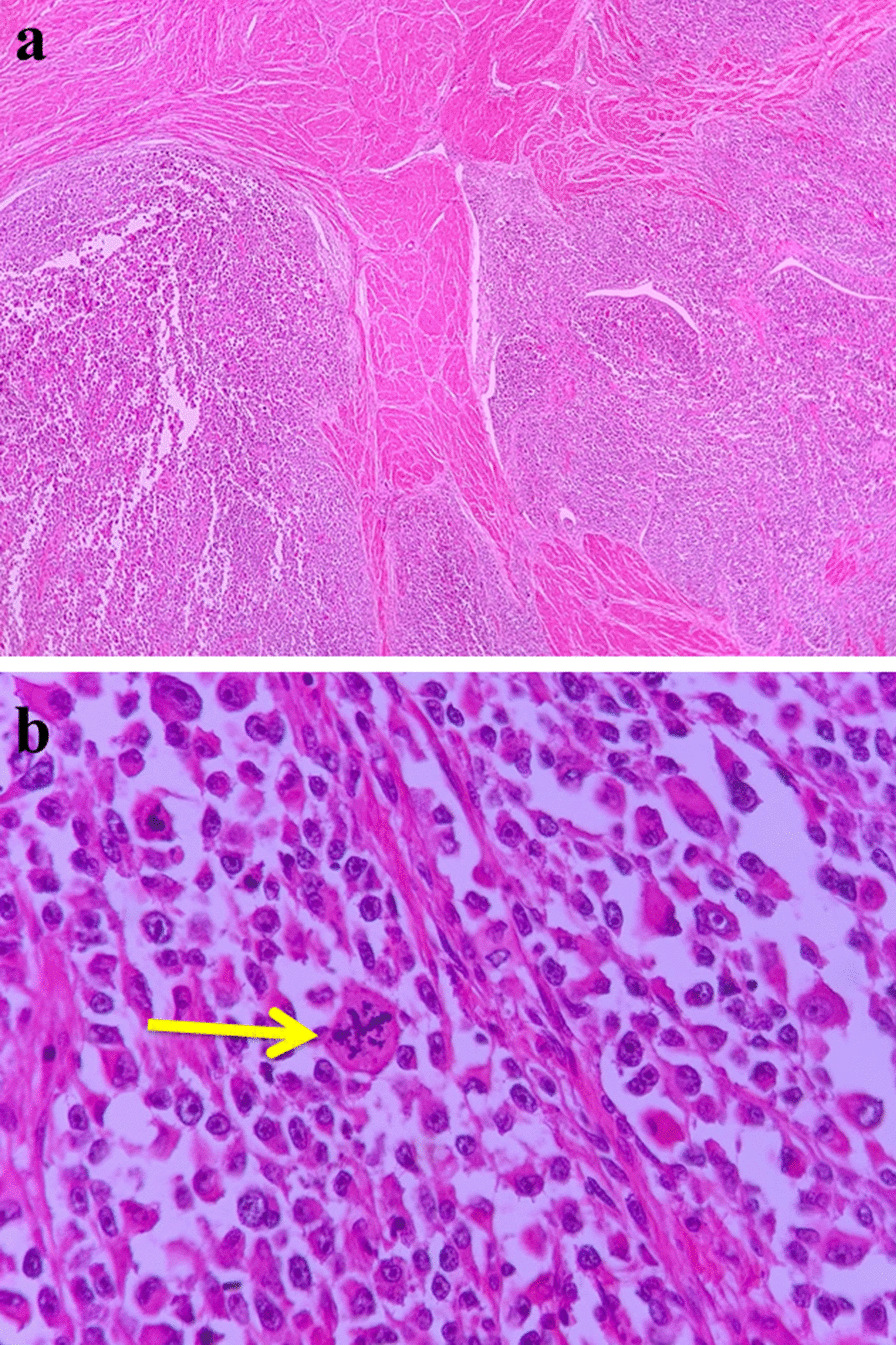
**a** Infiltration of tumor within myometrium. **b** Rhabdoid cells with eosinophilic brightly abundant cytoplasm and numerous mitotic figures (yellow arrow)

Grossly, the tumor was cream-colored and fleshy, measuring 10 × 8 × 6 cm, filling entire endometrial cavity, and protruding into endocervical canal (Fig. 3). Microscopically, the tumor was placed in endometrium with infiltrative border, and the myometrial invasion was less than 50%. In high-power microscopic field, rhabdoid cells were present with abundant bright eosinophilic cytoplasm and atypical enlarged nucleus (Fig. 4a). The tumor displayed variable degrees of necrosis, and tumor giant cells were present. Neither epithelial nor other heterologous component were seen. Numerous mitotic figures, including atypical forms and solid sheets of cells separated with fibrotic bands, were present (Fig. 4b).

**Fig. 5:**
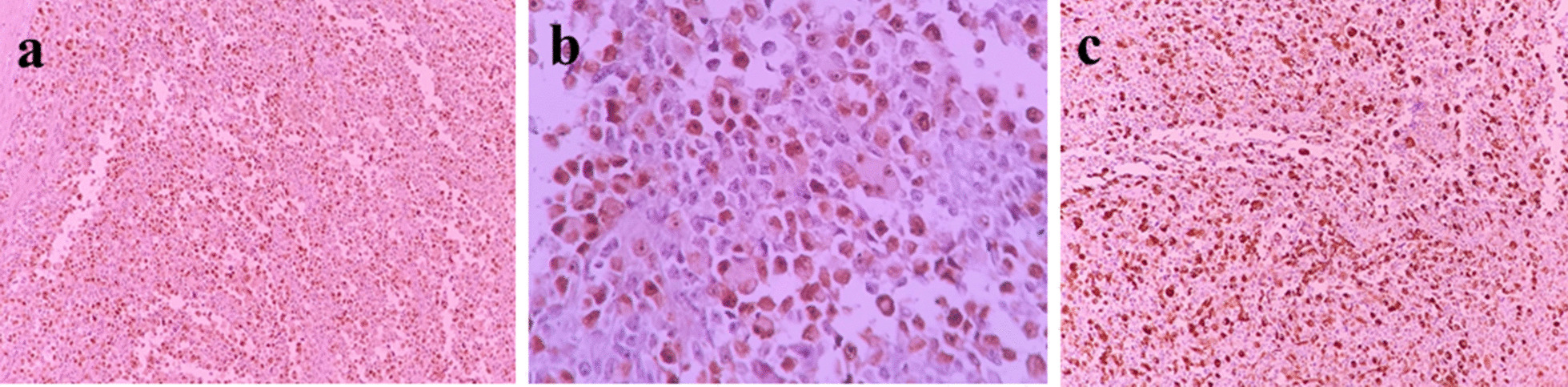
**a** Positive for desmin IHC staining in tumor cells. **b** Positive for MyoD1 IHC staining in tumor cells. **c** Positive for myogenin IHC staining in tumor cells

These histologic patterns and cytological features describe the solid variant of alveolar subtypes of RMS. There was no lymphovascular space invasion. All 14 pelvic lymph nodes, cervix, vaginal margins, adnexa parameters, and peritoneal washing fluid were free of tumor. Immunohistochemistry (IHC) test showed positive desmin (Fig. 5a), MyoD1 (Fig. 5b), and myogenin (Fig. 5c) cytoplasmic staining.

## Discussion

Uterine sarcoma accounts for 3–7% of uterine malignant neoplasms and is more aggressive in comparison with epithelial neoplasms [4, 6].Uterine sarcomas are classified as homologous, including elements that are normally found in the uterus (endometrial stromal sarcoma, undifferentiated sarcoma, fibrosarcoma, leiomyosarcoma) or heterologous, including sarcomatous components that are not usually found in the uterus (rhabdomyosarcoma , chondrosarcoma, osteosarcoma, liposarcoma). RMS is the most common soft tissue malignancy in children, and a remarkable majority of genital tract RMSs occur in vagina in infants and adolescents [2].Pure heterologous RMS of uterine corpus is rare in adults. Prognosis and survival rate of RMS is disappointing in adult patients.

We hereby presented a case of uterine alveolar RMS in a 60-year-old postmenopausal female patient. We searched PubMed and Scopus for English original articles, letters, and short communications with keywords: “Rhabdomyosarcoma” AND “Uterus” since 1972. Articles that reported rhabdomyosarcoma in corpus of uterus in adults were reviewed if their full text was available. Table 1 summarizes a series of these cases from medical literature. These articles reported 41 patients between 22 and 90 years of age of which 21 patients (51.2%) had pleomorphic type, 9 patients (21.9%) had embryonal type, and 6 patients (14.6%) had alveolar type RMS. The tumor size ranged from 5 to 20 cm. Different management approaches were considered for each patient, including surgery and adjuvant and/or neoadjuvant chemotherapies. Thirty-one patients (75.6%) had undergone total hysterectomy (TH) + bilateral salpingoophorectomy (BSO) (2 subtotal hysterectomy), and 19 patients (46.3%) had received adjuvant and/or neoadjuvant treatment with chemotherapy and/or irradiation. Also, 21 patients (53%) died within a mean duration of 12.9 months after initial diagnosis or therapy.

**Table 1:** Management and outcomes in women with uterine corpus RMS

	References	RMS type	Age	Tumor size	Treatment	Outcome
1	Oluwole	Pleomorphic	51 years	7 × 5.8 × 3.4 cm	TH + BSO + RPLND + resection of gross tumors	Death due to disease 0.5 months
	Fadare *et al*. [16]		74 years	14 × 6.5 × 5.1 cm	TH + BSO + PPLND + resection of gross tumors	Lost to F/U
			79	10 × 6 × 4 cm	TH + BSO + PPLND + resection of gross tumors + adj pelvic RT	Death due to disease 6.3 months
			68 years	9.3 × 7.4 × 4.2 cm	TH + BSO + PPLND + resection of gross tumors + adj pelvic RT + VAC chemotherapy	Death due to disease 19 months
2	Sohsuke Yamada *et al*.	Embryonal	55 years	11 × 7 cm	TH + BSO	6 month F/U free of disease
3	Garrett La *et al*.	Embryonal	28 years	5.6 cm	TH + omentectomy + VAC chemotherapy	40 week F/U during chemo
4	Masaharu Fukunaga	Alveolar	72 years	6 cm	TH + BSO + PLND + resection of gross tumors + adj chemotherapy	Death due to disease 12 months
5	Masashi Takano *et al*.	Embryonal	76 years	15 × 17 cm	TH + BSO + adj chemotherapy	10 months F/U after surgery
6	Carolina Chmaj *et al*.	Pleomorphic	66 years	8.2 × 6.4 cm	TH + BSO + adj chemotherapy	Death 2.5 y/s after surgery
7	Donkers *et al*.	Embryonal	90 years	450 gram uterus	TH + BSO	Death 7 months after surgery probably due to CHF
		Pleomorphic	56	150 gruterus	TH + BSO + neoadj and adj chemoradiation	Death 6.5 y/s after surgery
8	Roberto Chiarle	Alveolar	80 years	9 cm	TH + BSO + adj radiotherapy	Death 4 months after surgery
9	Hideo Teshima *et al*.	Embryonal	52	320 gruterus	TH + BSO + PLND + adj chemotherapy	30 month F/U after surgery
10	Fabrice Leung *et al*.	Pleomorphic	68 years	5 × 5 × 2 cm	TH + BSO	12 month F/U after surgery
11	Reynolds *et al*.	Embryonal	65 years	27 × 17 × 15 cm	TH + BSO + partial cystectomy + omentectomy + PLND	Death 10 days after surgery
12	Siegal *et al*.	Unavailable	69 years	6.1 × 5 × 4.9 cm	TH + BSO + PPLND + resection of gross tumors + adj pelvic RT	F/U 3 months after surgery
13	Pinto *et al*. [5]	Alveolar	40 years	12 cm	Chemotherapy	18 month F/U after surgery
		Pleomorphic	68 years	13.6 cm	Chemotherapy	Death 10 months after surgery
		Pleomorphic	65 years	Unavailable	Chemotherapy	26 month F/U after surgery
		Pleomorphic	62 years	15.2 cm	Hospice care	Death 4 months after surgery
		Pleomorphic	70 years	13 cm	Chemotherapy	9 month F/U after surgery
		Alveolar	64 years	14.5 cm	Hospice care	Death 6 weeks after surgery
14	Sara Alavi *et al*.	Pleomorphic	73 years	6.5 × 6 × 5 cm	TH + BSO	Unavailable
15	Norio Motoda *et al*.	Alveolar	50 years	10 cm	Partial resection	Death 19 days after surgery
16	Afshan Ambreen *et al*.	Embryonal	22	8 × 10 cm	TH + BSO + adj chemoradiation	Death 14 months after surgery
17	Aljehani *et al*.	Embryonal	54 years	10 cm	Palliative pelvic radiotherapy	Unavailable/new case
18	Goldstein *et al*.	Unavailable	73 years	Unavailable	TH + BSO + adj chemotherapy	11 month F/U after surgery
19	Ashley S. Case *et al.*	Alveolar	21	10.2 × 8.2 cm	TH + BSO + neoadj and adj chemotherapy	20 month F/U after surgery
20	Podczaski *et al*.	Pleomorphic	73 years	10 × 7 × 5 cm	TH + BSO + omentectomy + PPLND + adj pelvic radiation	Death 3 months after surgery
21	Dae Woon Kim *et al*.	Spindle cell	76 years	20 × 15 × 7 cm	Subtotal hysterectomy + BSO	Death 3 months after diagnosis
22	Mutsumi Kuroki *et al*.	Unavailable	36 years	Unavailable	Chemotherapy	Death 5 months after diagnosis
23	Okada *et al*. [7]	Pleomorphic	53 years	15 cm	Radical hysterectomy + adj radiation	Recurrence 2 months after surgery
24	Mccluggage *et al*.	Spindle cell	28 years	12 cm	Modified radical hysterectomy + PLND + adj chemoradiation	F/U 2 years after surgery
		Pleomorphic	67 years	5 cm	Subtotal hysterectomy + BSO	Death 3 days after surgery
25	Hart *et al*. [17]	Embryonal	22 years	100 gruterus	Radical hysterectomy + adj and neoadj chemotherapy VAC	F/U 4.4 years after surgery
		Pleomorphic	70 years	450 gruterus	TH + BSO	Death 4 months after surgery
		Pleomorphic	56 years	Unavailable	Palliative chemotherapy	Death 6 weeks after diagnosis
26	Kevin Holcomb *et al*.	Pleomorphic	63 years	6 × 6 × 2 cm	TH + BSO	Death 4 years after surgery
27	S. Yeasmin *et al*.	Pleomorphic	60 years	Unavailable	TH + BSO + PPLND + adj chemoradiation	Death 20 months after surgery
28	Jaworski *et al*.	Pleomorphic	71 years	1200 gram uterus	TH + BSO	F/U 7 months after surgery
29	Katalin Borka *et al*.	Pleomorphic	67 years	15 cm	TH + BSO + PPLND + adj chemotherapy	F/U 12 months after surgery
	Aminimoghaddam *et al*.	Alveolar	60 years	10 × 8 × 6 cm	TH + BSO + PPLND + neoadj chemotherapy + adj pelvic irradiation	Alive at 12 month F/U

*TH* total hysterectomy, *BSO* bilateral salpingoophorectomy, *PLND* pelvic lymph node dissection, *PPLND* pelvic and paraaortic lymph node dissection, *adj* adjuvant, *neoadj* neoadjuvant, *F/U* follow-up, *CHF* chronic heart failure

Previous pelvic irradiation and long-term use of tamoxifen are among the risk factors for developing uterine RMS [7]. Our patient did not have these risk factors, but she had history of fracture in lower extremities due to osteopetrosis twice, while none of these cases had similar comorbidity and no correlation was found between RMS and osteopetrosis.

The histologic differential diagnosis of uterine RMS is endometrial stromal sarcoma, leiomyosarcoma, adenosarcoma/carcinosarcoma. As we mentioned earlier, diagnosis of pure RMS in uterus involves precise histopathological evaluation. Widespread and perfect sampling is necessary to uncover any epithelial component. IHC test can help us to evaluate any suspected areas [5]. IHC staining identifies muscle-specific proteins such as actin, myosin, myoglobin, desmin, and MyoD1. Rhabdomyosarcoma is classified into four main subtypes: embryonal, alveolar, spindle cell/sclerosing, and pleomorphic. Myoglobin and desmin are positive in most RMS cases, while epithelial markers are not detectable [8]. Fluorescent *in situ* hybridization (FISH) and reverse-transcription polymerase chain reaction (RT-PCR) methods can detect fusion genes such as PAX3_FOXO 1 and PAX7_FOXO1 that are found in alveolar RMS [9]. IHC staining was positive for cytoplasmic desmin, MyoD1, and myoglobin in this patient, but cytogenetic tests were not available.

Ferguson *et al*. studied 15 women with gynecologic RMS and showed that the most prevalent site of the gynecologic RMS is uterine cervix, followed by uterine corpus. In this study, the 5-year disease-specific survival (DSS) was 29%, and older age, widespread disease at the time of diagnosis, and unfavorable histologic variants such as pleomorphic and alveolar led to poor outcomes in adults [10].

Enzo Ricciardi *et al*. studied 15 patients with primary cervical RMS . Vaginal bleeding and pelvic mass were the most common symptoms, and Intergroup Rhabdomyosarcoma Study Group (IRS) clinical staging was the best predictor of prognosis [11]. This case presented with heavy vaginal bleeding and a large protruding cervical mass.

There is limited experience with RMS treatment in adults, and a combined modality is considered based on IRS group studies in younger patients [12]. Complete excision of localized tumor, if feasible, followed by chemotherapy or radiotherapy is a common approach.

Maha *et al*. evaluated 137 patients with nonmetastatic gynecologic RMS, in a systematic review. Surgery was the main approach for local control of tumor in all patients [13]. According to the suggested scheme in this study, our patient was in the high-risk group owing to a large bulky pelvic mass, heavy vaginal bleeding, and alveolar variant of RMS. Therefore, we chose neoadjuvant chemotherapy (NACT) followed by definitive surgery.

In a retrospective analysis of 171 patients (older than 18 years of age), Ferrari *et al*. concluded that RMS responds to chemotherapy in adults exactly as it does in children, and there is no reason to select different approaches [14].

Luca Bergamaschi *et al*. reported 95 adult patients with RMS in a prospective single-center case series. The treatment recommendation was a multidisciplinary approach that included surgery, chemotherapy, and radiotherapy. Chemotherapy was recommended for all patients. It consisted of a multidrug treatment, alternating the ifosfamide, vincristine, and actinomycin-D (IVA) regimen with ifosfamide, vincristine, and adriamycin (IVAd) or ifosfamide, vincristine, and etoposide (IVE). Delayed surgery was performed after three to four courses of chemotherapy. Radiotherapy (with a conventional fractionation and doses ranging from 50 to 60 Gy) was suggested in all cases of alveolar, not otherwise specified (NOS) RMS, and embryonal RMSs that were incompletely resected at diagnosis. In this study, the 5-year event-free and overall survival rates were 33.6% and 40.3%, respectively [15]

We used neoadjuvant chemotherapy, including VAC regimen, for the patient, and she received external pelvic irradiation with a total dose of 5040 cGy within 6 weeks after surgery.

## Conclusion

RMS of uterine corpus is rare in adults. It is an aggressive tumor with a poor outcome, despite multidisciplinary approach. Histopathological criteria, IHC staining, and molecular studies differentiate RMS from the other uterine sarcomas. More studies are needed on treatment options in adults.

## Data Availability

All the data of this case are available. **Ethics approval and consent to participate** The patient has signed an informed consent form for all the diagnostic and therapeutic plans.

## Abbreviations

RMS: Rhabdomyosarcoma
CT Scan: Computerized tomography
BSO: Bilateral salpingoophorectomy
PLND: Pelvic lymph node dissection
IHC: Immunohistochemistry
TH: Total hysterectomy
FISH: Fluorescent *in situ* hybridization
RT-PCR: Reverse-transcription polymerase chain reaction
NACT: Neoadjuvant chemotherapy
IVA: Ifosfamide, vincristine, actinomycin-D
IVAd: Ifosfamide, vincristine, adriamycin
IVE: Ifosfamide, vincristine, etoposide
VAC: Vincristine, actinomycin-D, cyclophosphamide
DSS: Disease-specific survival
IRS: Intergroup Rhabdomyosarcoma Study Group
F/U: Follow-up
CHF: Chronic heart failure
